# Emergency Department Readmission Rate within 72 Hours after Discharge; a Letter to Editor

**Published:** 2017-04-03

**Authors:** Hassan Barzegari, Mohammad Ali Fahimi, Schwann Dehghanian

**Affiliations:** ^1.^ Department of Emergency Medicine, Golestan General Hospital, Ahvaz Jundishapur University of Medical Sciences, Ahvaz, Iran.

**Keywords:** Emergency service, hospital, patient readmission, practice management

There are several factors that lead to early return of patients to the medical centers. These could be due to latent illnesses, misdiagnosis or even inadequate medical care due to staff being worn out (1, 2). Determining the mentioned factors could help decrease the rate of readmission, restore patient's confidence in health centers and decrease the cost of medical care. 

Chi lung Wu et al. in 2010 sought to find out the major cause of patients’ return within 24 hours after discharge from emergency department (ED) and showed that abdominal pain and fever were the most common causes of patients' return, respectively. They found that, on average, 5.5% of discharged patients returned to the hospital (3). 

Tazhibi et al. carried out a research to find out major causes of readmission in Alzahra Hospital, Isfahan, Iran, and concluded that factors related to the patient and hospital could be held responsible for the patients’ readmission to the studied hospital (1). 

Verelst S et al., investigating the reasons for patient re-hospitalization within 72 hours after discharge in a teaching hospital in Belgium, found that 1.9% of admissions to the ED were in fact readmissions. 12% of this population were readmitted because of misdiagnosis, (2). 

Chang SY et al. carried out a research in a teaching hospital in Taiwan to investigate the reasons for non-traumatic patient readmission within 72 hours of their discharge. They reported the rate of 3.5% for their readmissions out of their overall 72000 visits. Misdiagnosis was the most common problem among patients who had been readmitted shortly after discharge (4). 

In a retrospective cross-sectional study, we evaluated the medical profiles of all patients who were readmitted within 72 hours of their discharge from the ED of Golestan Hospital, Ahvaz, Iran, during July to December 2013, aiming to find the readmission rate of this ED. 63736 patients were admitted to the ED during the 6-month study period, 635 (0.99 %) cases of which were readmitted within the first 72 hours of discharge (67.1% male). 338 (53.2%) were 16 – 45 years old. Traumatic injuries (37.1%), flank pain (8.3%), acute coronary syndrome (7.2%), Cerebro-vascular accident (5.2%), and abdominal pain (4.8%) were among the most frequent causes of previous ED visit of readmitted patients. Finally, 70.2% of these patients were discharged again, 15.7% were admitted to other hospital wards, and 14.1% were transferred to other hospitals.

Comparing the results of the present study with previous ones indicates that readmission rate can vary widely not only among different countries but also among various hospitals of the same country. For instance, in the studied hospital, which is a trauma center in the region, readmission rate of trauma patients is higher.

Since readmissions cause unnecessary overcrowding in ED, it would be best if each hospital evaluated their rate of readmission and its causes, and then tried to relieve the problems found. This can be effective in better management of ED, reduction of treatment costs, increasing patient satisfaction, and prevention of ED overcrowding.
